# Psychosocial and psychological interventions for schizophrenia relapse prevention: A bibliometric analysis

**DOI:** 10.1017/gmh.2024.49

**Published:** 2024-04-12

**Authors:** Fang Liu, Wei Qiao, Xiuxia Yuan, Gangrui Hei, Xin Li, Yu Zhang, Xueqin Song, Dongqing Zhang

**Affiliations:** 1 Department of Operation Management, The First Affiliated Hospital of Zhengzhou University, Zhengzhou, Henan Province, China; 2 Department of Psychiatry, The First Affiliated Hospital of Zhengzhou University, Zhengzhou, Henan Province, China; 3 Department of Logistics, Shengjing Hospital of China Medical University, Shenyang, Liaoning Province, China

**Keywords:** schizophrenia, psychosocial and psychological interventions, relapse prevention, bibliometric analysis

## Abstract

Various psychosocial and psychological interventions have been developed to reduce schizophrenia relapse prevention. A better understanding of these active interventions is important for clinical practice and for meaningful allocation of resources. However, no bibliometric analysis of this area has been conducted. Studies were retrieved from the Web of Science Core Collection database. The publication outputs and cooperation of institutions were visualized with Origin 2021. Global cooperation was visualized using ArcGIS Pro3.0. VOSviewer was used to generate visualizations of network of authors and keywords. The number of annual publications generally showed a fluctuating upward trend over the past 20 years. Germany published the most relevant articles (361, 26.76%). The Technical University of Munich was the most productive institution (70, 9.86%). Leucht Stefan published the most articles (46, 6.48%) and had the highest number of citations (4,375 citations). *Schizophrenia Research* published the most studies (39, 5.49%). Keywords were roughly classified into three clusters: cognitive behavioral therapy (CBT), family interventions and family psychoeducation and other factors related to interventions. The findings provided the current status of research on psychosocial and psychological interventions for schizophrenia relapse prevention from a bibliometric perspective. Recent research has mainly focused on CBT, family interventions and family psychoeducation.

## Graphical abstract

**Figure:**
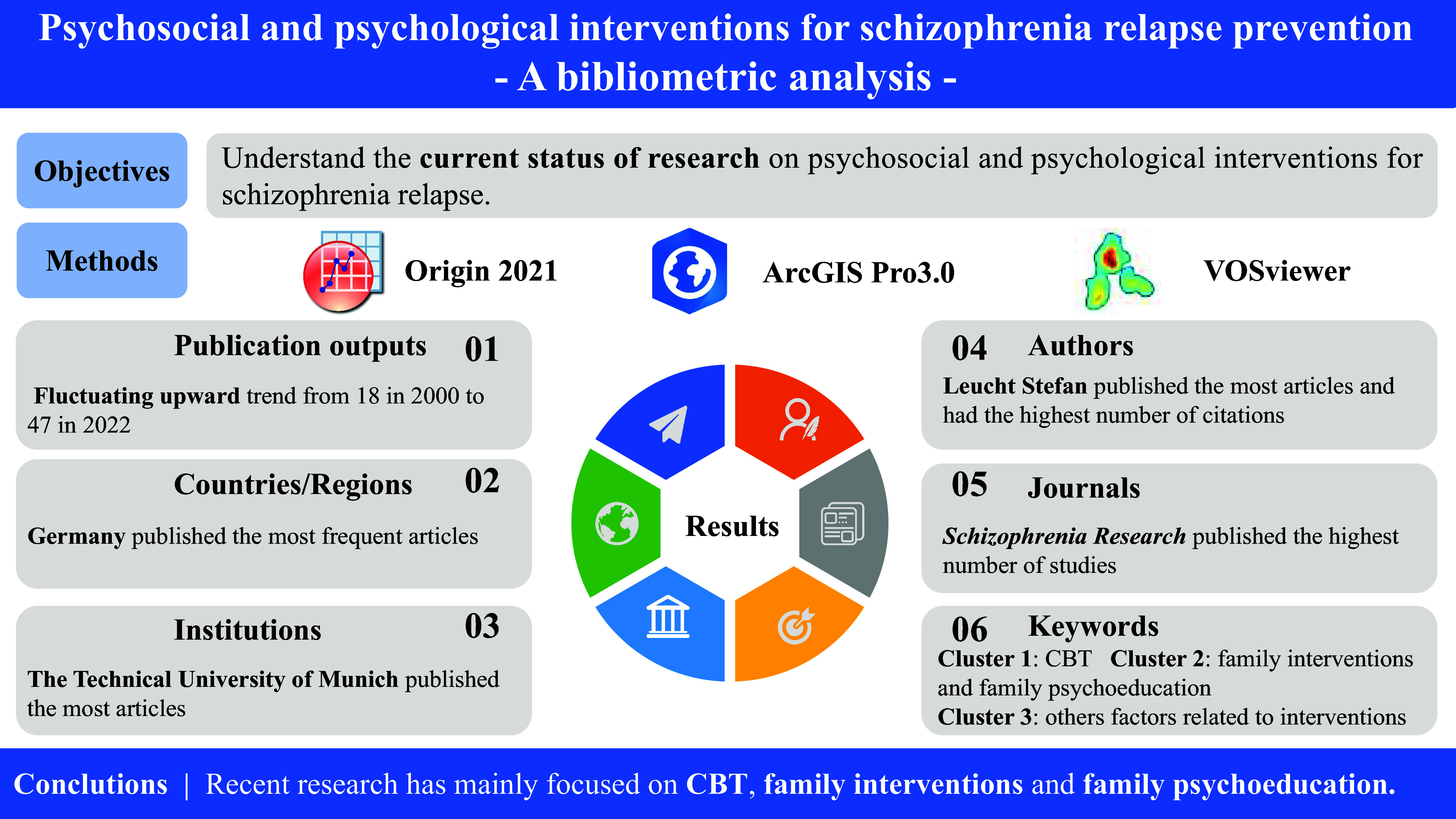

## Impact statements

The clinical course of schizophrenia is often characterized by recurrent relapses. Various psychosocial and psychological interventions have been developed to further reduce the relapse. A better understanding of these active interventions is important for clinical practice and resource allocation. This study sought to understand the current status of research on psychosocial and psychological interventions for schizophrenia relapse prevention from a bibliometric perspective. The field is gradually maturing and has the potential for further development. Recent research has mainly focused on cognitive behavioral therapy, family interventions and family psychoeducation. These findings may serve as a navigation map for clinicians and researchers to identify key information for future research.

## Introduction

Schizophrenia is a psychiatric disorder characterized by psychotic symptoms (hallucinations, delusions and disorganized speech); negative symptoms (such as decreased motivation and diminished expressiveness) and cognitive deficits (impaired executive functions, memory and speed of mental processing) (Marder et al., 2019). It typically emerges in late adolescence or young adulthood, persisting throughout life (Schmitt et al., 2023). The global age-standardized point prevalence of schizophrenia in 2016 was estimated to be 0.28% (Charlson et al., 2018). In China, the weighted lifetime prevalence is as high as 0.6% (Huang et al., 2019). Schizophrenia is one of the mental disorders with the highest mortality risk. The rate of all-cause mortality in those with schizophrenia was 2.08 times that of the general population over a 15-year follow-up period (Hayes et al., 2018), with a weighted average of 14.5 years of potential life lost (Hjorthøj et al., 2017). Another meta-analysis of 135 cohort studies observed a 2.9-fold increased all-cause mortality in patients with schizophrenia versus the general population and a 1.6-fold increased risk versus physical disease-matched general population controls (Correll et al., 2022).

The clinical course of schizophrenia is often characterized by recurrent relapses (Miller et al., 2023). A broad definition of relapse included when symptom exacerbation occurs alone, and when it impairs an individual’s functioning, as well as hospitalization (Taylor and Jauhar, 2019). In the pharmacological maintenance trials, 24% of patients taking maintenance antipsychotics relapsed within a year, important differences among the drugs (Schneider-Thoma et al., 2022). That is, one in four patients might experience relapse within a year despite well-supervised maintenance pharmacotherapy (Furukawa and Bighelli, 2022). Relapses can lead to risk of harm, loss of autonomy and substantial distress for individuals and families (Ceraso et al., 2020). These individuals who have a relapsing course of illness are less likely to marry or sustain long relationships (Taylor and Jauhar, 2019) and are linked to an increased risk of suicidal behavior (Rina et al., 2011). Additionally, relapse may carry a biological risk that active psychosis reflects a period of disease progression insofar as patients may not return to their previous level of function and treatment refractoriness may emerge (Emsley et al., 2013).

Various psychosocial and psychological interventions have been developed to further reduce the relapse (Bighelli et al., 2021); these interventions may work in tandem with pharmacologic treatments, synergistically alleviating symptoms and increasing functioning (Faden and Citrome, 2023). These interventions include, but are not limited to: psychoeducation (Xia et al., 2011), family interventions (Pharoah et al., 2010), community-based rehabilitation interventions (Ye et al., 2023), cognitive behavioral therapy (CBT) (Tai and Turkington, 2009; Grant et al., 2012), cognitive remediation therapy (CRT) (Puig et al., 2014), combined motivational interviewing and CBT (MI-CBT) (Reddy et al., 2023), social skills programs (Almerie et al., 2015), recovery-oriented cognitive therapy (CT-R) (Zhang and Perivoliotis, 2022), vocational interventions (VI) (Singh et al., 2021) and Open Dialog (Buus et al., 2019). Evidence revealed that ORs compared with treatment as usual and corresponding percentages of participants who relapsed were 0.35 (95% CI 0.24–0.52) with 16% for family interventions, 0.33 (0.14–0.79) with 15% for relapse prevention programs, 0.45 (0.27–0.75) with 20% for CBT, 0.56 (0.39–0.82) with 23% for family psychoeducation, 0.62 (0.44–0.87) with 25% for integrated interventions and 0.63 (0.42–0.94) with 25% for patient psychoeducation. By contrast, 35% of participants receiving treatment as usual relapsed (Bighelli et al., 2021).

A better understanding of these active interventions is important for clinical practice and resource allocation. Moreover, given the rapid increase in publications over recent decades, new approaches are needed to review and analyze trends within knowledge domains. Bibliometrics is an ideal and effective tool that offers a quantitative approach for analyzing a large body of work and identifying the central research topics and intellectual structures in a research area (Andersen and Lund, 2020). As a broad synthesis method, it answers the question of ‘what is studied’ and provides an integrated conceptual and methodological toolbox to support the new field of meta-research (Sabe et al., 2023). Currently, it is widely employed and acknowledged as an important tool in research evaluations (Zhiguo et al., 2023). Several analyses have investigated the publication trends in the domain of schizophrenia from a bibliometric perspective. For instance, Seda Kiraz et al. revealed the global scientific outputs of schizophrenia publications (Kiraz and Demir, 2021). Michel Sabe et al. provided a holistic summary of changes in negative symptoms in schizophrenia research (Sabe et al., 2023). Antonia Najas-Garcia et al. analyzed the trends of motivation in schizophrenia (Najas-Garcia et al., 2018). Other studies have explored the influences of inflammation (Sun et al., 2022), oxidative stress (Chen et al., 2023) and gut microbiota (Yang et al., 2022) in schizophrenia. Only one study provided a comprehensive overview of the use of CBT for schizophrenia (Fei et al., 2021). However, CBT is only one kind of non-pharmacological intervention. Therefore, bibliometric analysis of comprehensive psychosocial and psychological interventions for schizophrenia is lacking.

In this context, we sought to understand the current status of research on psychosocial and psychological interventions for schizophrenia relapse prevention to provide clinicians, researchers and policymakers with the research network. To achieve this goal, we systematically evaluated information on the distribution of publications and cooperation across countries, institutions, authors, journals and analyzed the keywords used to present trends in this field from a bibliometric perspective.

## Methods

### Search strategy and data collection

**Figure 1.**
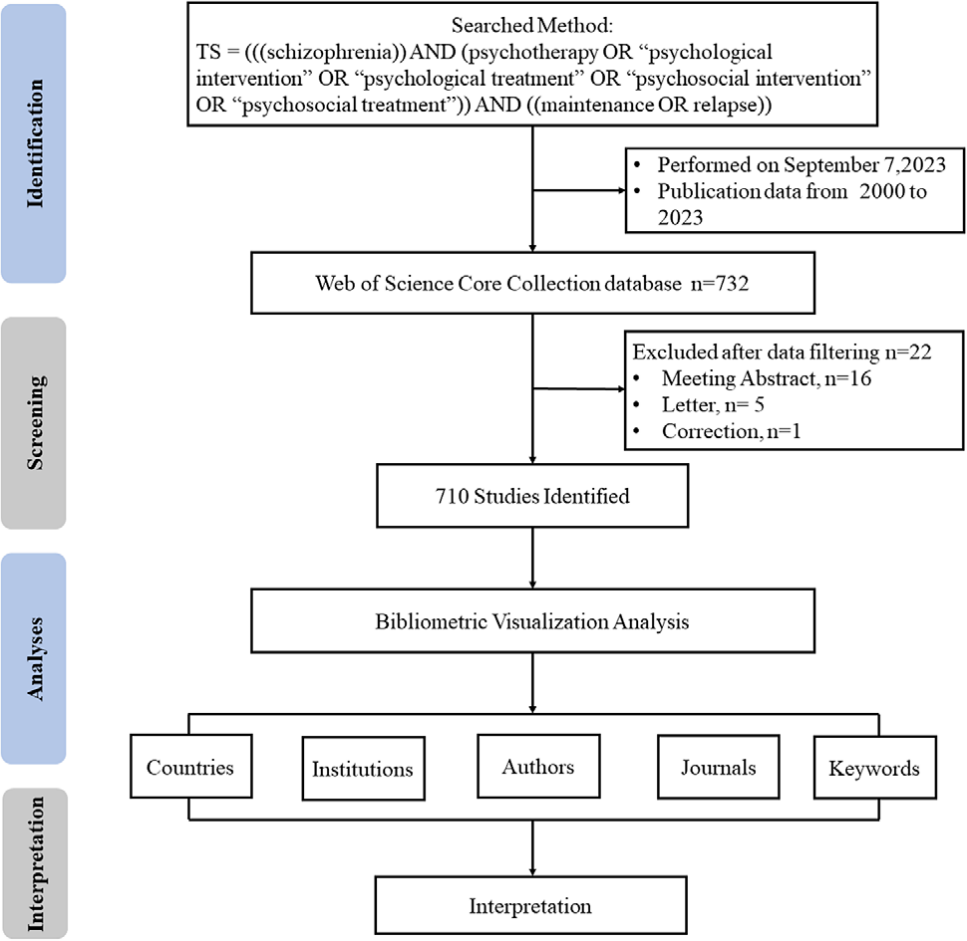
Flowchart of the bibliometric analysis.

Studies were retrieved from the Web of Science (WOS) Core Collection database. The WOS is the most frequently used database for bibliometric research, as it includes a high number of scientific publications and provides overall data sources for bibliometric software (Xu et al., 2021). Publications included in the WOS database can be representative of research in the discipline (Zhiguo et al., 2023). The search terms were as follows: (((schizophrenia)) AND (psychotherapy OR “psychological intervention” OR “psychological treatment” OR “psychosocial intervention” OR “psychosocial treatment”)) AND ((maintenance OR relapse)). The time span was 2000–2023. The data were independently collected by two authors (LF and ZY); any discrepancies were taken for reassessment by a third-party. All data searches and downloads were performed on September 7, 2023. We excluded conference abstracts, letters and corrections (n = 22), resulting in a final selection of 710 articles. The full record of the included publications was downloaded in the form of a TXT file. The following data were collected from each publication: year of publication, country/region, institution, author, journal and keywords. The search strategy is shown in Figure 1.

### Data analysis

In this study, Origin 2021, ArcGIS Pro3.0 and VOSviewer were used for data analysis and visualization. Figures displaying publication outputs and cooperation of institutions were generated with Origin 2021. The global distribution of publications and cooperation among countries/regions were visualized using ArcGIS Pro3.0. VOSviewer (version 1.6.18, https://www.vosviewer.com/) was used to generate networks to visualize the associations of authors and keywords. It is a program that can generate bibliometric maps, which are mainly used to visualize cooperation and co-citation networks (van Eck and Waltman, 2010). In these visualizations, a node represents an item (i.e., an author or keyword), the size of the node reflects its importance (i.e., the frequency of co-authorship or the number of publications) and links represent collaborations (Chen et al., 2023).

## Results

### Publication outputs

**Figure 2.**
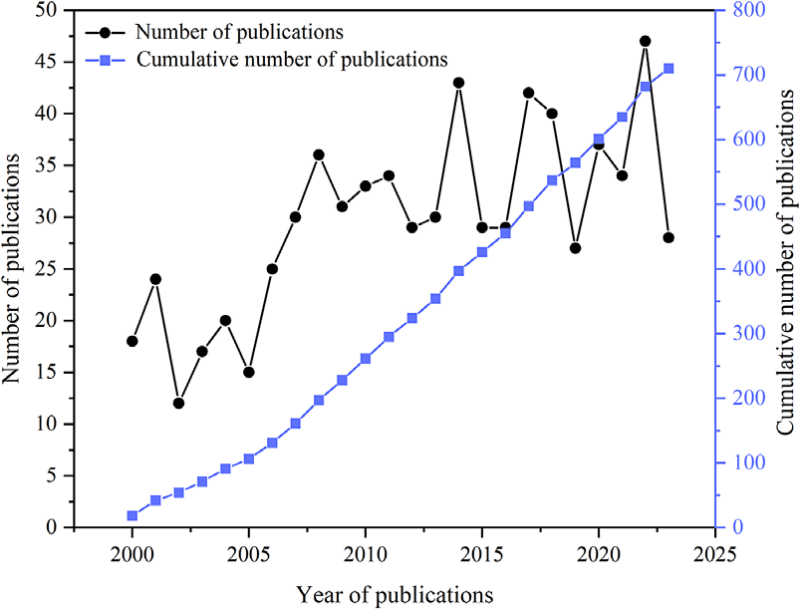
Trends in the number of relevant publications from 2000 to 2023.

To evaluate the current status of research on psychosocial and psychological interventions for schizophrenia relapse prevention, we first aimed to determine changes in the number of publications each year by calculating the number of studies published from 2000 to 2023. Over the past 20 years, the number of annual publications has generally showed a fluctuating upward trend, from 18 in 2000 to 47 in 2022, with an annual growth rate of 4.46% (excluding 2023, because the statistical data for the year of 2023 were not complete by the data of data extraction: September 7, 2023) (Figure 2).

### Distribution of publications and cooperation among countries/regions

**Figure 3.**
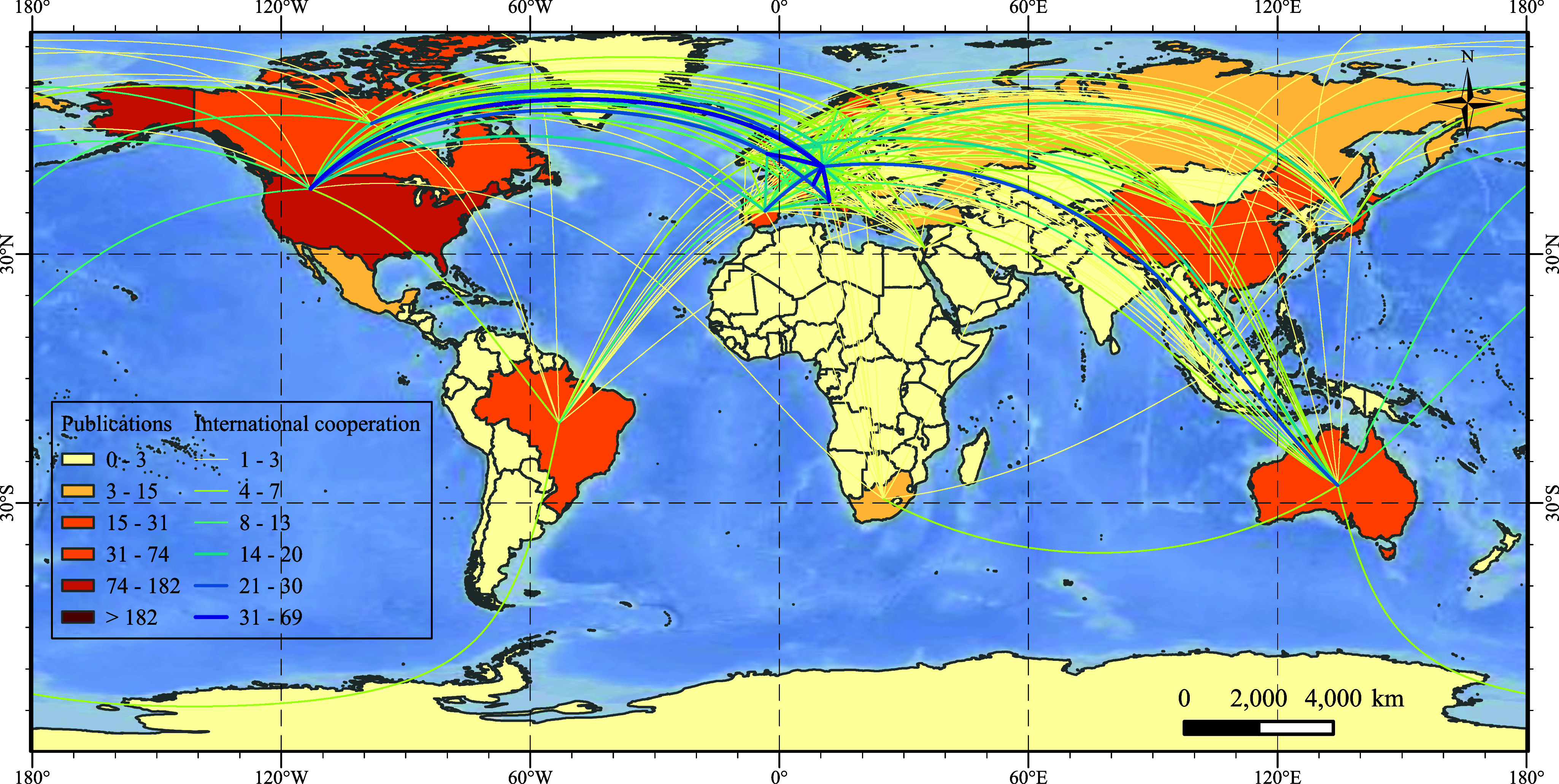
Distribution of publications and cooperation among countries/regions. *Notes:* The colors of the countries represent the number of publications. Lines represent the cooperation between countries/regions, while the line colors indicate the frequency of cooperation.

After examining the publication output, we next presented the distribution of publications and cooperation among countries/regions. A sum of 61 countries/regions contributed to the publication output, with 40 countries/regions (65.57%) with fewer than 10 publications. Germany published the most frequent articles (361, 26.76%), followed by the United States (182, 13.49%) and the United Kingdom (114, 8.45%). The most frequent international cooperation was between Germany and the United States (frequency = 69), followed by Germany and Switzerland (frequency = 46) and between the United Kingdom and Germany (frequency = 40) (Figure 3).

### Distribution of publications and cooperation among institutions

**Figure 4.**
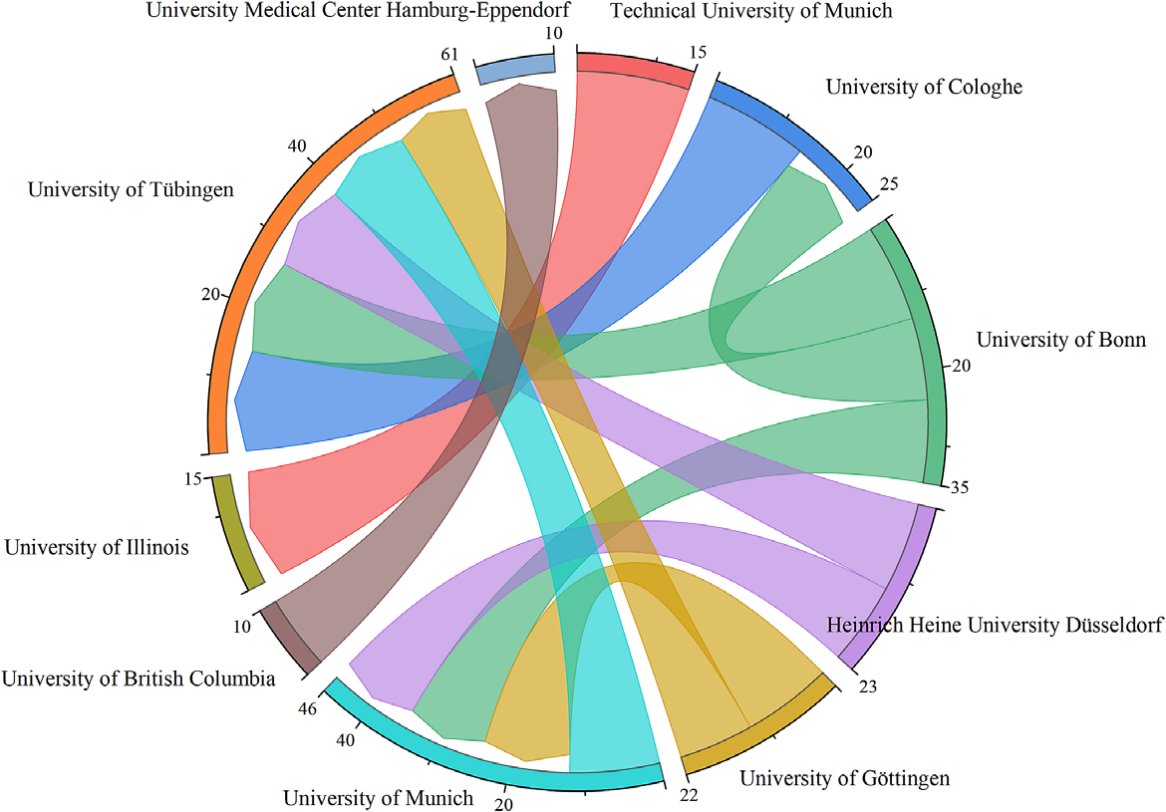
Chordal graph of institutions with at least 10 relevant cooperation.

After examining the publication output and cooperation at the global level, we next examined these variables at the institutional level. A total of 1,000 institutions published research in this field, while 654 (65.40%) research institutions had only a volume of one study. The top five productive institutions were in Germany. The Technical University of Munich published the most articles (70, 9.86%), followed by the University of Tübingen (39, 5.49%), the University Medical Center Hamburg-Eppendorf (36, 5.07%), the University of Hamburg (31, 4.37%) and Heidelberg University (30, 4.23%). Figure 4 shows the institutions with at least 10 relevant cooperations. The most frequent cooperation was between the Technical University of Munich (Germany) and the University of Illinois Urbana-Champaign (USA) (frequency = 15), followed by the University of Cologne and the University of Tübingen (both in Germany) (frequency = 14), as well as the University of Bonn and the University of Tübingen (both in Germany) (frequency = 13).

### Distribution of publications and cooperation among authors

**Figure 5.**
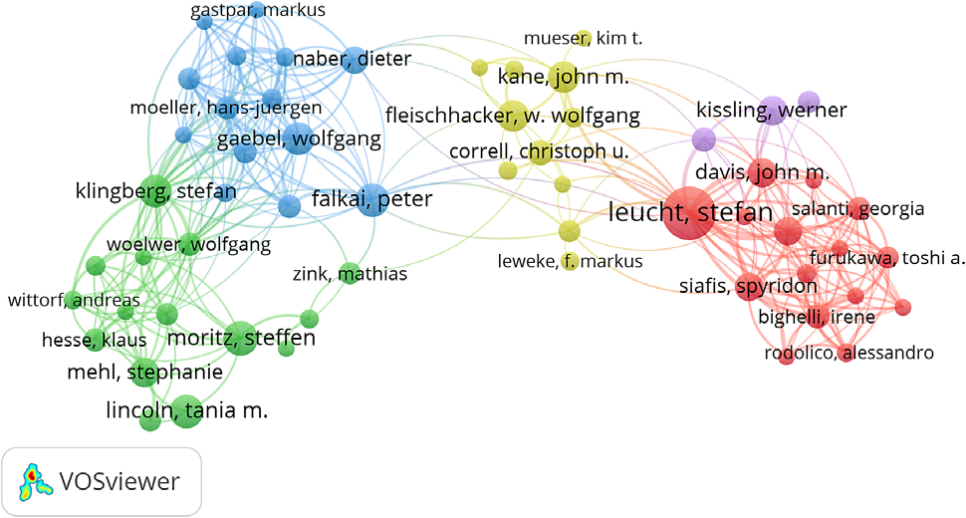
Cooperation network of core authors. *Notes:* Each node represents an author. The node size represents the number of publications. Colors indicate clusters.

After examining the publication output and cooperation at the global and institutional levels, we next presented the authors. A total of 2,930 researchers have published articles in this field; among them, 2,588 authors (86.62%) published only one study. According to Price’s Law, the quantity of papers published by the least prolific author among the core authors is equal to 0.749 times the number of papers published by the most prolific authors (Yu et al., 2022). It could be calculated that authors who published at least five relevant articles could be identified as core authors in this area; we identified 58 (1.98%) core authors, with a total of 556 articles (78.31%). These core authors formed five cooperation networks (Figure 5). Table 1 shows the top 10 most prolific authors in this area, 7 of whom were from Germany. Leucht Stefan published the most articles (46, 6.48%) and had the highest number of citations (4,375 citations).

**Table 1.** Top 10 most prolific authors in the field

Rank	Author	Institution	Country	Publications, n (%)	Citations
1	Leucht Stefan	Technical University of Munich	Germany	46 (6.48)	4,375
2	Moritz Steffen	University of Hamburg	Germany	20 (2.82)	584
3	Falkai Peter	University of Munich	Germany	19 (2.68)	686
4	Lincoln Tania M.	University of Marburg	Germany	19 (2.68)	527
5	Klingberg Stefan	University of Tübingen	Germany	18 (2.54)	484
6	Gaebel Wolfgang	Heinrich Heine University	Germany	17 (2.39)	661
7	Fleischhacker W. Wolfgang	Medical University of Innsbruck	Austria	16 (2.25)	774
8	Kane John M	Feinstein Institutes for Medical Research	USA	16 (2.25)	1,479
9	Davis John M	University of Chicago	USA	14 (1.97)	2,528
10	Kissling Werner	Technical University of Munich	Germany	14 (1.97)	1,327

### Distribution of journals

After examining the publication output and cooperation at the global, institutional and author levels, we next presented journals. Articles in this field have been published in 221 journals. Table 2 shows the top 10 journals in terms of publication frequency, accounting for 30.42% of the total publications. *Schizophrenia Research* published the highest number of studies (39, 5.49%), followed by *Schizophrenia Bulletin* (33, 4.65%) and *European Archives of Psychiatry and Clinical Neuroscience* (27, 3.80%). Based on Journal Citation Reports (JCRs) from 2022, four journals were placed in Quartile 1 (Q1), while five were placed in Quartile 2 (Q2).

**Table 2.** Top 10 journals that published the most articles

Rank	Journal	Country	Publications, n (%)	Citations	IF in 2022	JCR quartile
1	*Schizophrenia Research*	Netherlands	39 (5.49)	2,109	4.5	Q2
2	*Schizophrenia Bulletin*	USA	33 (4.65)	2,405	6.6	Q1
3	*European Archives of Psychiatry and Clinical Neuroscience*	Germany	27 (3.80)	620	4.7	Q1
4	*Cochrane Database of Systematic Reviews*	UK	24 (3.38)	1,534	8.4	Q1
5	*Frontiers in Psychiatry*	Switzerland	18 (2.54)	94	4.7	Q2
6	*Journal of Clinical Psychiatry*	USA	18 (2.54)	1,518	5.3	Q1
7	*BMC Psychiatry*	UK	17 (2.39)	187	4.4	Q2
8	*Nervenarzt*	Germany	15 (2.11)	106	1.1	Q4
9	*Psychology and Psychotherapy–Theory, Research and Practice*	UK	13 (1.83)	160	3.4	Q2
10	*Clinical Psychology & Psychotherapy*	UK	12 (1.69)	163	3.6	Q2

**Abbreviations:** IF, impact factor; JCR, Journal Citation Reports; Q1, Quartile 1; Q2, Quartile 2; Q4, Quartile 4.

### Keyword co-occurrence analysis

**Figure 6.**
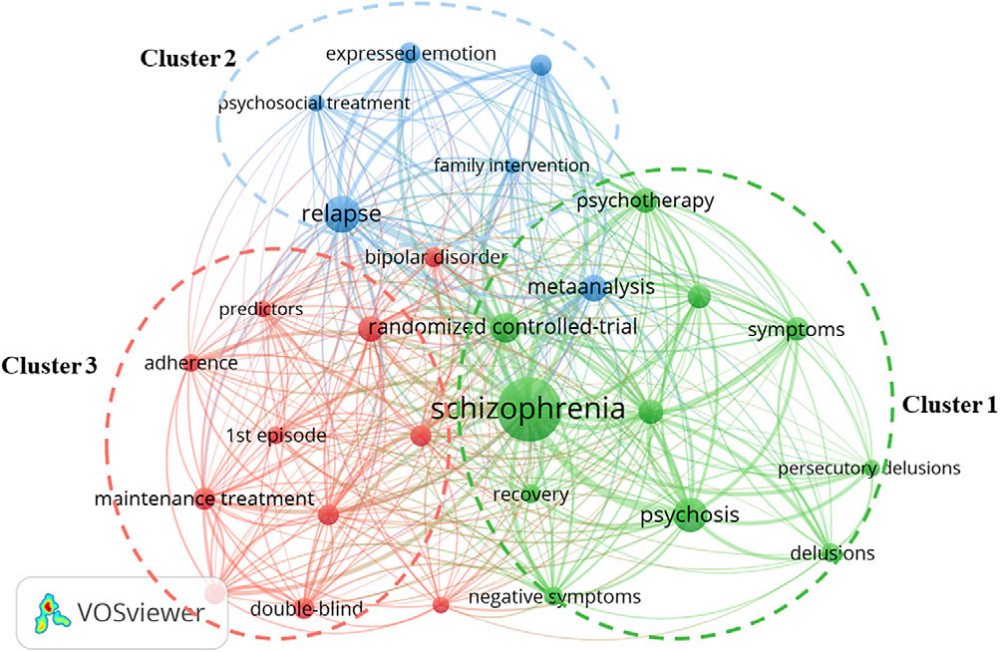
Map of keyword co-occurrence based on keywords with at least 30 occurrences. *Notes:* Node size reflects the frequency of keyword occurrence. Colors indicated clusters.

The publication output, cooperation at the global, institutional and author levels and journals were introduced in the previous section; this section details keyword co-occurrence. A total of 36 keywords appeared at least 30 times; they were roughly classified into three clusters based on the number of articles in which they co-occurred (Figure 6). Cluster 1 focused on the CBT and included terms such as “cognitive-behavioral therapy”, “negative symptoms”, “persecutory delusions”, “psychosis”, “psychotherapy”, “randomized controlled-trial, recovery” and “symptoms”. In Cluster 2, the high-frequency keywords were “expressed emotion”, “family intervention”, “follow-up”, “family psychoeducation”, “psychosocial treatment” and “relapse”; this cluster focused on the family interventions and family psychoeducation. Cluster 3 included other factors related to interventions, with a high frequency of the following keywords: “first episode”, “adherence”, “antipsychotics”, “maintenance treatment”, “predictors” and “quality-of-life”.

## Discussion

Psychosocial and psychological interventions are integral components in the treatment of schizophrenia in addition to pharmacotherapy (Faden and Citrome, 2023). This bibliometric analysis examined the current status of research on psychosocial and psychological interventions for schizophrenia relapse prevention. The analyses indicated that the number of publications has fluctuating increased during the past 20 years, which means that the field is gradually maturing and has the potential for further development. Recent research has mainly focused on CBT, family interventions and family psychoeducation.

The author’s country reflects the scientific output, academic climate and the degree of importance of a subject area in that country. From a geographical perspective, studies have been published in diverse countries with a broad distribution. However, most of the countries had few publications, which highlighted the importance of global research cooperation. Germany has made the largest contribution to global research in terms of the number of publications, followed by the United States and the United Kingdom, which indicates that they are the principal countries in the field. This is in line with previous studies reporting a significant correlation between article productivity and economic indicators, such that developed countries or countries with high economic productivity also tend to publish the most articles (Kiraz and Demir, 2021). In addition, countries with more publications have more frequent information exchanges and cooperation, indicating that international academic collaborations may foster research outputs.

The publishing institution reflects whether the institution is part of the main research force on a subject matter. In terms of institutions, more than half of the research institutions had only a volume of one study, indicating a lack of continuity of research in this field, similar to the findings of Fu et al. (2023). Besides, the top five most productive institutions were all in Germany, corresponding to the country with the most publications worldwide, and demonstrating that conducting advanced research is critical to the academic level of a country (Zhao et al., 2022). The Technical University of Munich published the most articles. This may mean that it has the highest scientific productivity in this domain. In addition, similar to the country analysis results, institutions that had more publications also had more cooperation.

Similar author analyses can help identify the core authors and major collaborations in the field. Among authors with relevant work, the majority of authors (86.62%) had published only one study, indicating that most scholars conducted only experimental research and had not researched further and that their research lacked continuity. On the other hand, it also proved that the breadth of participation in the study emphasized the high level of global interest in this critical area. Only 1.97% were identified as core authors, indicating that the majority of individuals demonstrated interest in this area but had not conducted substantial relevant research, and their research continuity and opportunities for cooperation remained limited. Notably, since core authors had published 78.31% of the total publications, it appears that the core author group is stable and plays an essential role in this domain. Additionally, the authors with the most publications were identified; Leucht Stefan (from Germany) was the most productive author, suggesting that these productive authors have made outstanding contributions to the field, and their future in-depth research is expected to provide the latest information in this field (Zhao et al., 2022; Zhang et al., 2023).

Journals are important conveyers of scientific research and scholarly communication. The top 10 most active journals published approximately 30% of the total publications. Among them, *Schizophrenia Research* was the most productive journal, consistent with findings from other bibliometric analyses on schizophrenia-related oxidative stress (Chen et al., 2023), CBT (Fei et al., 2021) and negative symptoms (Sabe et al., 2023). Among the top 10 most active journals, nine journals were of high quality (Q1 or Q2), indicating that the level and quality of research is high and that readers can follow these journals to understand the academic dynamics of this domain.

Keywords with high frequency are often used to summarize the main theme, reveal the knowledge foundation and structure and identify cutting-edge perspectives of research domains (Fang et al., 2023). Of mention, this study is not an exhaustive description of every detail, but rather provides a macro perspective through methodological integration to support scientific judgment of trends in psychosocial and psychological interventions for schizophrenia relapse prevention. In our study, keyword co-occurrence was roughly divided the whole network into three clusters, with each cluster focused on a main topic. Cluster 1 focused on the CBT, and Cluster 2 focused on the family interventions and family psychoeducation. This may indicate that these three forms are the most important and common types of psychosocial and psychological interventions for schizophrenia relapse prevention. This resonates with the research by Bighelli et al. (2021). They analyzed 20 psychosocial and psychological interventions reported in 72 randomized controlled trials containing 10,364 participants and found robust benefits of family interventions, family psychoeducation and CBT in terms of reducing the risk of relapse. It also illustrates the rationality of our analysis to a certain degree.

CBT is a psychological intervention that aims to change the way in which a person interprets and evaluates their experiences, helping them to identify and link feelings and patterns of thinking that underpin distress (Guaiana et al., 2022). However, it is debated whether CBT is effective against schizophrenia relapse. As Bighelli et al. pointed out in their 2021 study, CBT is efficacious in reducing relapse and in improving many secondary outcomes (overall, positive and negative symptoms; adherence and functioning) (Bighelli et al., 2021). While Jauhar et al. directly critique one aspect of the methods employed by Bighelli et al. and believed that describing the benefits of CBT against relapse as “robust” could be considered overly strong (Jauhar et al., 2022). And Jauhar et al. concluded that CBT does not prevent relapse based on the present evidence (Jauhar et al., 2019). In addition, there is no clear and convincing advantage for CBT over other psychosocial therapies for people with schizophrenia (Jones et al., 2018). Therefore, more high-quality trials are needed to reach a conclusion regarding whether CBT is effective for relapse prevention.

Additionally, families play a critical role in providing care and support for persons living with schizophrenia (Clari et al., 2022). Thus, many different models of family interventions have been developed. Evidence has confirmed that almost all family intervention models are efficacious in preventing schizophrenia relapse (Rodolico et al., 2022). Of these models, the simplest form (family psychoeducation) was ranked among the most efficacious interventions for relapse prevention (Rodolico et al., 2022). Policymakers and clinicians should consider prioritizing family interventions and family psychoeducation when allocating resources, especially in low-resource settings, and planning maintenance treatment for patients with schizophrenia.

In recent years, new topics have become more relevant and are currently being implemented in a growing number of services across the globe, such as Open Dialog. Open Dialog approaches encourage active participation of families and social networks and emphasize genuine collaboration within highly integrated systems of health-care service delivery (Buus et al., 2021). Evidence revealed that Open Dialog approach might generate psychosocial resources that are not provided through conventional psychiatric treatment programs and could augment the outcomes of such treatment programs (Buus et al., 2019). Due to the relatively small volume of literature, they were not present in the cluster.

In addition, multiple factors related to interventions, such as object (first episode); treatment (maintenance treatment) and outcome (quality of life, adherence), were included in Cluster 3. A combination of systemic antipsychotic maintenance treatment and psychosocial and psychological interventions for first-episode drug-naive schizophrenia patients can help improve their prognosis and increase the possibility of recovery. Quality of life and adherence are important indices for evaluating secondary efficacy outcomes of interventions (Bighelli et al., 2021).

### Limitations

Our bibliometric analysis had some limitations. First, the data were retrieved from the WOS Core Collection database alone, which may not include all articles. However, the WOS database is considered the optimum database for bibliometric studies as the use of additional biomedical databases does not significantly increase the yield of relevant journals (Aggarwal et al., 2016). Second, the WOS Core Collection database only includes studies published in English, which may lead to selection bias. Notably, the extraction of themes was only based on the keyword co-occurrence analysis, which may overlook fine-grained information. In addition, the protocol of our study did not distinguish between the types of schizophrenia. For the group of resistant schizophrenia, psychosocial interventions and psychotherapy are very important. Future studies could consider a differential analysis to gather a more detailed answer to research questions.

## Conclusions

This study provides the current status of research on psychosocial and psychological interventions for schizophrenia relapse prevention from a bibliometric perspective. The field is gradually maturing and has the potential for further development. Recent research has mainly focused on CBT, family interventions and family psychoeducation. These findings may serve as a navigation map for clinicians and researchers to identify key information for future research.

## Data Availability

The original data are available upon request to the corresponding author.

## Abbreviations

CBT: cognitive behavioral therapy
CRT: cognitive remediation therapy
MI-CBT: combined motivational interviewing and cognitive-behavioral therapy
CT-R: recovery-oriented cognitive therapy
IF: Impact Factor
JCR: Journal Citation Reports
Q1: Quartile 1
Q2: Quartile2
Q4: Quartile4
