# Supplementary material for: Psychosocial and psychological interventions for schizophrenia relapse prevention: A bibliometric analysis
Source: Glob Ment Health (Camb). 2024 Apr 12;11:e49. doi: 10.1017/gmh.2024.49 (PMC11058533; doi:10.1017/gmh.2024.49)
Supplement: Peer review history

# Editor report: Recommendation: Psychosocial and psychological interventions for schizophrenia relapse prevention: A bibliometric analysis — R0/PR2

**DOI:** 10.1017/gmh.2024.49.pr2

**Affiliations:** Division of Behavioral Health Services and Policies, Columbia University Vagelos College of Physicians and Surgeons, United States

No accompanying comment.

# Editor report: Decision: Psychosocial and psychological interventions for schizophrenia relapse prevention: A bibliometric analysis — R0/PR3

**DOI:** 10.1017/gmh.2024.49.pr3

**Affiliations:** Johns Hopkins University Bloomberg School of Public Health, United States

No accompanying comment.

# Editor report: Recommendation: Psychosocial and psychological interventions for schizophrenia relapse prevention: A bibliometric analysis — R1/PR5

**DOI:** 10.1017/gmh.2024.49.pr5

**Affiliations:** Division of Behavioral Health Services and Policies, Columbia University Vagelos College of Physicians and Surgeons, United States

No accompanying comment.

# Editor report: Decision: Psychosocial and psychological interventions for schizophrenia relapse prevention: A bibliometric analysis — R1/PR6

**DOI:** 10.1017/gmh.2024.49.pr6

**Affiliations:** Johns Hopkins University Bloomberg School of Public Health, United States

No accompanying comment.
